# Immersive Virtual Reality Reaction Time Test and Relationship with the Risk of Falling in Parkinson’s Disease

**DOI:** 10.3390/s23094529

**Published:** 2023-05-06

**Authors:** Pablo Campo-Prieto, José Mª Cancela-Carral, Gustavo Rodríguez-Fuentes

**Affiliations:** 1 Faculty of Physiotherapy, Department of Functional Biology and Health Sciences, University of Vigo, HealthyFit Research Group, Galicia Sur Health Research Institute (IIS Galicia Sur), SERGAS-UVIGO, E-36005 Pontevedra, Spain; pcampo@uvigo.es (P.C.-P.); gfuentes@uvigo.es (G.R.-F.); 2 Faculty of Education and Sports Science, Department of Special Didactics, University of Vigo, HealthyFit Research Group, Galicia Sur Health Research Institute (IIS Galicia Sur), SERGAS-UVIGO, E-36005 Pontevedra, Spain

**Keywords:** virtual reality exposure therapy, digital health, Parkinson’s disease, reaction time, falls, videogames, physical activity, measurement of movement, rehabilitation, games for health

## Abstract

Immersive virtual reality (IVR) uses customized and advanced software and hardware to create a digital 3D reality in which all of the user’s senses are stimulated with computer-generated sensations and feedback. This technology is a promising tool that has already proven useful in Parkinson’s disease (PD). The risk of falls is very high in people with PD, and reaction times and processing speed may be markers of postural instability and functionality, cognitive impairment and disease progression. An exploratory study was conducted to explore the feasibility of reaction time tests performed in IVR as predictors of falls. A total of 26 volunteers (79.2% male; 69.73 ± 6.32 years) diagnosed with PD (1.54 ± 0.90 H&Y stage; 26.92 ± 2.64 MMSE) took part in the study. IVR intervention was feasible, with no adverse effects (no Simulator Sickness Questionnaire symptoms). IVR reaction times were related (Spearman’s rho) to functionality (timed up and go test (TUG) (rho = 0.537, *p* = 0.005); TUG-Cognitive (rho = 0.576, *p* = 0.020); cognitive impairment mini mental state exam (MMSE) (rho = −0.576, *p* = 0.002)) and the years of the patients (rho = 0.399, *p* = 0.043) but not with the first PD symptom or disease stage. IVR test is a complementary assessment tool that may contribute to preventing falls in the proposed sample. Additionally, based on the relationship between TUG and reaction times, a cut-off time is suggested that would be effective at predicting the risk of suffering a fall in PD patients using a simple and quick IVR test.

## 1. Introduction

Of all the neurogenerative disorders that affect older people, Parkinson’s disease (PD) is the second most common and affects up to 2% of older people (65 years old and above) [1]. Motor symptoms, such as bradykinesia, tremor, rigidity, and balance disturbances [2] are characteristic of this disorder. PD is characterized not only by these motor aspects, but also by many non-motor symptoms including cognitive impairment, sensory abnormalities, behavioral changes, sleep disturbances, autonomic dysfunction, and some other symptoms which are more difficult to categorize, such as fatigue [3].

The cognitive impairment inherent in PD often leads to the slow processing of information. The symptoms of this, which can include deficits in processing speed and attention, cognitive inflexibility, and forgetfulness [4], may start to appear from the very first stages of the disease [5]. Reduced reaction times (RT) and processing speeds have been identified by some authors [6] as markers of postural instability and gait freezing, since such a reduction in speed is associated with difficulty in making turns [7]. When compared to their healthy peers, people with PD suffer a much higher risk of falling, with some prospective studies showing that between 45% and 68% of people with PD fall each year [8,9]. For this reason, the study of gait patterns with sensors in PD patients [10] and other pathological conditions [11] has been explored in some studies.

Further studies have suggested that balance is related to executive functions and attention, while functional mobility is related to cognitive impairment, verbal fluency, and attentional ability [12]. Cognitive requirements are necessary to maintain balance [13] and can be interfered with when attention is deviated or reduced [14]. Executive function [15] and processing speed [16] are two of the main cognitive functions correlated with postural stability.

Some physical tests—such as the TUG test created and validated by Podsiadlo and Richardson [17] to assess functional mobility and the risk of falls in the elderly—have been used in PD [18]. The TUG-Cognitive task test is based on the dual-task paradigm, aims to quantify the influence of the cognitive sphere on a common functional task [19] and has already been used successfully in PD [20]. It should be noted that while cognitive impairments lead to poor dual TUG scores, the nature of the cognitive deficit accounting for that is unknown. For this reason and to evaluate the cognitive requirements from the point of view of the integration and processing of information, other evaluation methods are proposed.

Simple reaction time (SRT) is considered to be a reliable measure of information processing speed [21], and is the time taken by a subject to produce an intentional response to the presentation of a reaction stimulus [22].

Choice reaction time (CRT) is used as a measure of visual/perceptual decision time and is related to the same processes as that involved in SRT, in addition to processing the uncertainty about when random stimuli will appear next, i.e., a decision process. Decision-processing has been used with PD patients as a measure of bradyphrenia [23].

Regarding this, in the past, some studies have explored SRT and CRT in PD without reaching concordant conclusions [24].

On the one hand, there is research which concludes that patients are slower in SRTs (such as in detection and interference tasks), and not in CRTs (e.g., in decision making, visual search, and interference control), which supports the theory of a possible general impairment of processing speed [25,26]. On the other hand, there are other studies which suggest that the increased difficulty of the task is the main cause of the increase in RT [27,28].

Based on these findings, some authors have carried out several attempts to separate the components that could influence information processing from others. For example, Sawamoto et al., 2002 [29], showed that motor deceleration could not explain the increased reaction time in PD participants by subtracting the motor time from the total RT, thus isolating the motor component from the cognitive component. Furthermore, Cooper et al., 1994 [27], subtracted the SRT from the CRT to analyze motor preprogramming and reached the same conclusion. Finally, in the work of Vlagsma et al., 2016 [30], it was found that patients with PD presented mental slowness, which could also be separated from motor slowness.

However, some controversy has arisen regarding the impact of motor impairment on the execution of computerized SRT task evaluation, as the response depends on how capable the subject is of producing keystrokes with their fingers. Computer-based finger tapping (FT) tasks are often used traditionally to evaluate motor fitness. The classic FT has been used many times to measure fine motor control in PD [31] and is an extremely effective indicator of bradykinesia, as correlated with the dopaminergic stimulation status [32].

In the light of the possible limitations indicated in the evaluation of reaction times through traditional methods in this population and the lack of specificity in some of the physical tests that evaluate balance and the risk of falls with dual tasks, it is suggested that a novel tool such as immersive virtual reality (IVR) could be useful. 

IVR uses advanced hardware and customized software to create a digital 3D reality in which multisensory stimulation occurs for all of the users by stimulating them by computer-generated sensations and feedback [33]. The VR environment can be non-immersive, semi-immersive and fully immersive [34], and the presence and immersion of the system increases from the use of a PC (non-immersive), large monitors (semi-immersive) or CAVE rooms or head-mounted displays (HMD) (fully immersive) [35]. Therefore, HMD represents one of the most immersive VR technologies and a promising exercise tool that—although is still in the early stages of development—has already proven useful in gait management in PD [36], even when combined with a treadmill [37], or when combined with a treadmill and anti-gravity aids [38], as this study was able to prove in a systematic revision [39]. The potential of this technology has also recently been flagged up in the context of health-promoting physical activity, both for people with PD [40] and for healthy older people, [41] in tasks designed to measure FT in parkinsonian disorders [42] or in fine motor training of the fingers [43]. More recently, it has also been tested to assess performance in several specific reaction time tests on mixed martials arts fighters [44].

Furthermore, this virtual test could facilitate the measurement of reaction times in the performance of functional tasks—tasks that require greater mobility of the upper limbs, which can be a key factor in the avoidance of possible falls—and is not limited to measuring a single finger movement.

However, to our knowledge, there have been no studies that assess the functional reaction time tests and fall risk in PD performed in IVR. This information could be invaluable, since a simple test that measures the reaction time to stimuli could evaluate the risk of falling in people with PD, help therapists to adapt their sessions according to the risk that each patient presents and maximize the safety of the treatments. With this in mind, the primary objective of our study was to explore the feasibility of functional reaction time tests performed in IVR. Secondly, correlations of reaction times with functionality, cognitive impairment and disease progression were explored.

## 2. Materials and Methods

### 2.1. Participants

This study was announced in the Parkinson’s Association of Vigo (Spain). An informative visit was made to the association to provide information to the center’s management and rehabilitation team about the study. The center disseminated the information among its members and the staff proposed 32 pre-candidates. Taking into account our previous research involving the use of IVR in people with PD [34,38], sample selection criteria were established (Table 1). Finally, a sample of 26 volunteers (79.2% male; 69.73 ± 6.32 years) diagnosed with PD (1.54 ± 0.90 H&Y stage; 26.92 ± 2.64 MMSE) and members of the Parkinson’s Association took part in the study.

The Ethics Committee of the Faculty of Physiotherapy at the University of Vigo Institutional Review Board (no. 205-2022-1) approved this study, and signed informed consent was given by all participants before it commenced.

### 2.2. Virtual Reality Device

**Figure 1:**
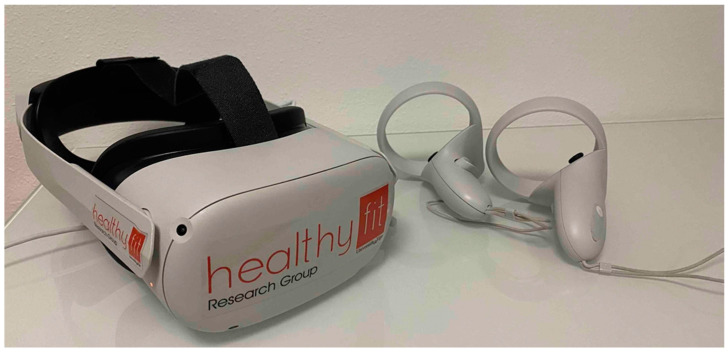
IVR Meta Quest II device used in the study; HMD with elite strap and controllers.

HMD Meta Quest II (Oculus VR, Menlo Park, CA, USA) was used. This system is standalone, requiring only 2 handsets and a Wi-Fi network for its use. The equipment used in this study included a more comfortable, ergonomic *Elite strap* which also contained an extra battery. An Apple iPad 10” was also provided so that the physical therapist could follow the performance of the task via the Oculus app more easily. The same hardware was employed with success in previous studies on martial art fighters [44] and in PD [40]. Figure 1 shows the equipment used.

### 2.3. Virtual Reality Software and Procedure

Based on our experience in the use of this technology, we decided to use the *Rezzil* player software available in the Oculus store (https://www.oculus.com, accessed on 15 November 2022). This software brings together a collection of games intended for sports training with the aim of improving performance in physical and cognitive abilities across a wide range of sports. This software was explored in another recent study [45].

For our study, we selected the *Reaction Wall* mode. In it, different sizes of walls are presented, where the goal is to respond to a random stimulus projected on a grid with a rapid movement of the upper limb (touching the appearing light with the hand). The larger the size of the wall, the greater the general movement that needs to be performed.

**Figure 2:**
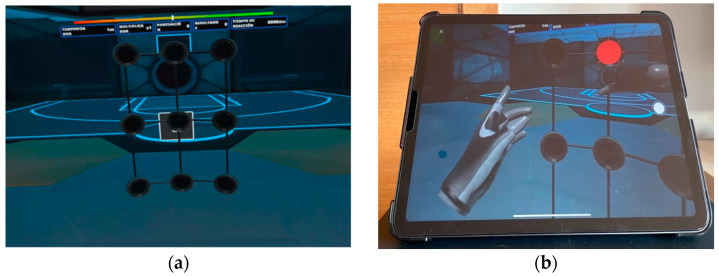
Micro wall mode of Rezzil game. Users must touch the red light as quickly as possible. (**a**) Screenshot of virtual scenario proposed for the test (3 × 3 grid) and placed in a virtual gym; (**b**) iPad and the view that the user and therapist are seeing simultaneously.

The options are *Micro wall*, *60º*, *180º* and *360º*. Under the criteria of the researchers and physical therapists with experience in the management of elderly people and PD, the *Micro wall* option was selected as it was considered more appropriate for the target group. It consists of a virtual 3 × 3 grid and random presentations of a stimulus in the form of a red light. The software measures the reaction time with an accuracy of 0.001 s (Figure 2).

**Figure 3:**
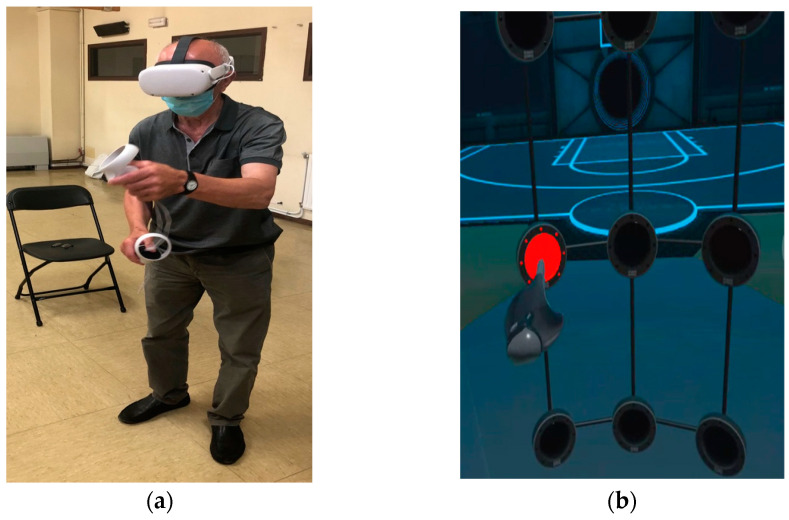
Participant during a session; (**a**) real movement and (**b**) virtual movement in the immersive scenario and red light as target.

The participants were given prior instructions by the physiotherapist as to what they were going to see in the IVR environment, and the task to be performed. All tests were supervised by the association’s physiotherapist. Each participant performed the test twice in a standing position, once to familiarize themselves with the virtual environment and the action to be performed in the test, and once as a measurement test. Participants performed a functional reaching gesture involving shoulder flexion and elbow extension. The task is determined by several multi-sensory cues: visual cues indicated by the presence of a red light to direct movement (this light is turned off when “touched”) and haptic and auditory cues with a brief vibration and sound when the target is reached (if the playing area is touched when there is no red light, the sound is different, and no vibration is produced). They made several attempts during a 60 s test and the fastest reaction time achieved was selected (Figure 3). At the end of the test, in a seated position, they were asked if they had experienced cybersickness.

### 2.4. Assessments

Taking into account the research objectives, the demographic and clinical characteristics of the patients were collected (sex, age, time since diagnosis, first symptoms, stage of the disease, presence of DBS surgery and falls in the last three months). The following aspects were also evaluated:

- Level of cognitive impairment. Cognitive ability was assessed with the mini-mental state examination (MMSE) scale [46]. The MMSE is a written test with a maximum score of 30, in which lower scores indicate more severe cognitive problems. The cut-off point established for the MMSE defines “normal” cognitive function is generally set at 24 and has been shown to be useful for the detection of dementia in older people [47].
- Functionality and functionality in dual tasks. These were evaluated with the classic timed up and go (TUG) test and a version of the same test with a cognitive component: the TUG-Cognitive task. All the participants completed both tests once. The TUG test was created and validated by Podsiadlo and Richardson [17] to assess functional mobility in the elderly. The test procedure consists of standing up from a seated position in a chair, walking a distance of 3 m, performing a 180 degree turn around a cone, then returning to the chair and sitting down again. The time taken to complete this task is recorded in seconds (taken by an evaluator using a stopwatch). The TUG-Cognitive task test is based on the dual-task paradigm, aims to quantify the influence of the cognitive sphere on a common functional task [19] and has already been used successfully in PD [20]. In our study, it was performed in the same way as the classic TUG test is, but this time the participants had to count down in threes from 51 while simultaneously performing the physical task (errors in the dual TUG answers were not considered a penalizing element in the test result).
- Risk of falling. This was evaluated with an ad hoc questionnaire about clinical characteristics (falls in the last three months) and according to the TUG test values following Podsiadlo and Richardson [17].
- Reaction time. This was evaluated with the *Rezzil player* software in the *Micro wall* mode available in the Oculus store (https://www.oculus.com, accessed on 15 November 2022). Participants had to react as quickly as possible to the random appearance of a red light on the presented grid. Participants made several attempts, and the best reaction time was recorded.
- The safety of the immersive experience was evaluated by the Simulator Sickness Questionnaire (SSQ), adapted and translated into Spanish [48]. The SSQ is an assessment tool used for recording the perceived severity of IVR simulator symptoms [49,50]. It comprises 16 items (e.g., nausea, headache, blurred vision, etc.) which are then divided into three subscales based on the type of symptoms experienced (nausea, oculomotor, and disorientation subscales). For each item, the severity of experienced discomfort is evaluated by the participant using a 4-point scale (1 for none, and 4 for severe).

All the evaluations were carried out on the same day. All patients were examined in the on state (dopaminergic medication) and did not experience wearing off symptoms. Demographic characteristics, level of impairment, functionality and risk of falling were recorded before the virtual test, and the reaction time and safety of the immersive exposure were recorded after the test was performed. Researchers from the University of Vigo, some of the authors of the study and the healthcare staff of the Association were in charge of the evaluations.

### 2.5. Statistical Analysis

Descriptive statistics were generated for all variables, stratifying the analysis into fallers and non-fallers. Distributions of variables were expressed as the mean ± standard deviation, as percentages, as a median and as minimum and maximum values. The normality of distribution for each variable was assessed by the Shapiro–Wilk test. The variables were not normally distributed (Shapiro–Wilk test, all *p* < 0.05). The homogeneity of the groups (non-fallers vs. fallers) was verified through the Mann–Whitney U test and chi-square test. Cut-off points (fallers vs. non fallers) were developed for the variables reaction time, TUG test and TUG-Cognitive test, using receiver operating characteristic (ROC) curve analysis. Spearman’s correlation coefficients were used to assess the degree of association between reaction time in relation to MMSE, TUG test, TUG cognitive test and age. The following standards were applied to interpret the agreement coefficients: 0 to 0.2 = very weak; 0.21 to 0.40 = weak; 0.41 to 0.60 = moderate; 0.61 to 0.80 = strong; 0.81 to 1.0 = very strong [51].

Statistical significance was set at *p* < 0.05. The data were analyzed using Statistical Package for the Social Sciences (SPSS Inc., Chicago, IL, USA) for Mac, version 25.0.

## 3. Results

The demographic and clinical characteristics of the patients are shown in Table 2. The sample was mainly composed of elderly male people (79.2%) with a normal cognitive status (26.92 ± 2.64 MMSE score) and with mild impairment (1.54 ± 0.90 H&Y stage). The assessment protocol was completed by all participants, and no adverse effects were observed during or after the virtual test (no SSQ symptoms).

**Figure 4:**
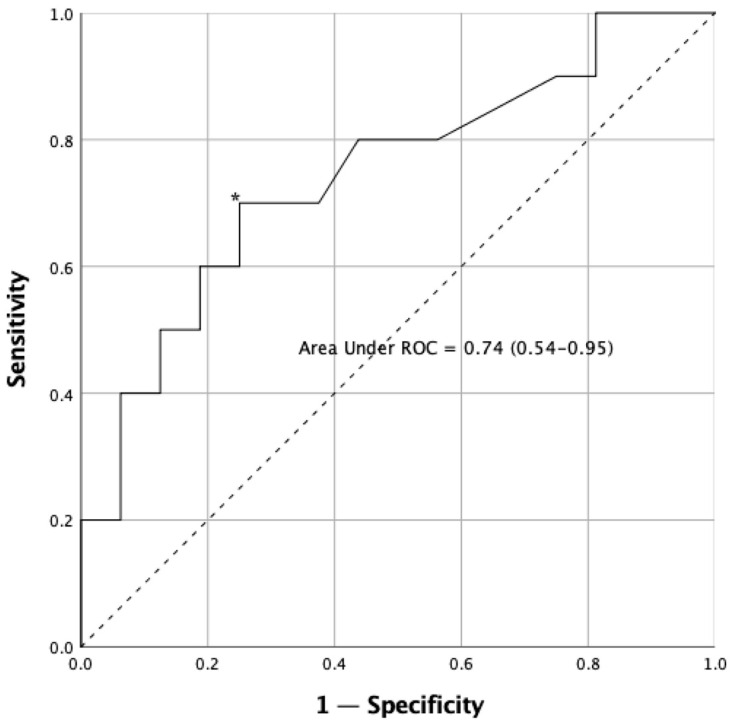
Receiver operating characteristic (ROC) curve for reaction time test in the sample. The asterisk (*) indicates the cut-off point that determines the highest joint sensitivity and specificity. The dashed line, running from point 0.0 to point 1.1 is called the reference diagonal, or line of non-discrimination. The solid line represents the cut-off points of the reaction time in relation to falls and provides information their respective sensitivity (Y axis) and 1—specificity (X axis). Both axes of the graph include values ranging from 0 to 1 (0–100%).

The analysis of the ROC curves, to establish cut-off points in the reaction time variable, TUG test and TUG-Cognitive test, is shown in Table 3. The results show that the variable that best discriminates between fallers and non-fallers is the TUG-Cognitive test score (AUC = 0.85). The reaction time variables regularly discriminate between falls in Parkinson’s disease (Figure 4), with the cut-off point being 0.574 s.

Table 4 shows the results of TUG, TUG-Cognitive, and reaction time in the sample (fallers vs. non-fallers). The ’non-fallers’ group achieved better results in all the tests. There are significant differences between groups in TUG times (*p* = 0.014), TUG-Cognitive times (*p* = 0.007) and virtual reaction times (*p* = 0.019).

Table 5 shows how reaction time is related to the cognitive status, functionality and age of the patients. The findings show a moderate correlation (0.41 to 0.60) in the case of MMSE scores, TUG, TUG-Cognitive times and non-fallers vs. fallers and a weak correlation with the age of the patients (0.21 to 0.40).

## 4. Discussion

Our findings are promising, showing that is feasible to carry out tests to measure the reaction times of people diagnosed with PD in immersive virtual reality environments. The proposed commercial hardware and software were shown to be safe and well-tolerated by the entire study sample (no cybersickness symptoms) in line with the findings demonstrated by Polechonski et al., 2022 [44], and Bagatin et al., 2022 [45], in studies on young athletes. 

The characteristics of the participants showed a mostly male sample with mild disease progression (1.54 ± 0.90, H&Y) situated in the older age range (69.73 ± 6.32 years) and with medium cognitive impairment (26.92 ± 2.64, MMSE). Furthermore, their functionality indicated some fall risks in the ‘fallers’ group (12.09 ± 5.27 s, TUG test), although it indicated no risk in the ‘non-fallers’ group (9.40 ± 1.15 s, TUG test). Therefore, further research is needed in which the samples are more diverse, focusing on people with greater disease progression, greater cognitive impairment or a lower level of functionality, so that the proposal can be tested in the other conditions that people with PD may present.

Deficits in information processing speed in PD have been investigated since the 1980s via reaction time tasks in order to establish whether slowness affects single cognitive mechanisms or whether it is a global impairment across all cognitive mechanisms [24,52,53]. More recent studies have concluded that slower information processing in PD is primarily associated with impaired motor processing speed and mechanisms of alert perception [54]. These cognitive problems could be objectified with our proposal; as previously mentioned, slower reaction times could be related to a higher risk of falls in PD [6] and in the future this could help to design new neurorehabilitation techniques, focusing on the improvement of perceptual and alertness mechanisms so as to improve balance components. This idea has already been suggested by some authors in studies of PD [55] and corroborated by others in studies of older people with cognitive impairment [56].

Our secondary objective was to explore the relationship between reaction times and cognitive impairment and functionality and disease progression. The significant relationships found between the results of the VRI tests and those of the conventional functional and cognitive evaluation tests confirm the relevance of this new tool in the assessment of fall risk.

- Reaction times and cognitive impairment: these were moderately negatively correlated with MMSE scores (rho = −0.576; *p* = 0.002) and as could be expected, people with greater cognitive impairment showed slower response times, in line with the findings of other studies [36].
- Reaction times and functionality: these were moderately positively correlated in both tests, with the classic TUG (rho = 0.537; *p* = 0.005) and with the cognitive TUG (rho = 0.454; *p* = 0.020). As already mentioned, balance and the risk of falling seem to be related to the ability to respond quickly to a stimulus. This fact coincides with that expressed in the study by Arroyo-Ferrer et al. [55], in which they found that balance measured through limits of stability (LOS) was related simply to reaction time as measured by a computer. In our study, a slower reaction time conditioned lower levels of functionality and a higher risk of falls in patients with PD. This fact reinforces the idea of introducing cognitive strategies into programs based on physical exercise as has been carried in other studies aimed at treatments for cognitive impairment [56], PD [57] and atypical Parkinson’s [58] in order to strengthen their therapeutic effects.
- Reaction times and disease progression: there was a positive moderate correlation of (rho = 0.456; *p* = 0.019) with the non-faller vs. faller condition and a positive weak correlation with the age of the patients (rho = 0.399; *p* = 0.043), although not with the first symptom or the stage of the disease. These results might seem contradictory since, under normal conditions, older age could lead to worse performance in the reaction time test due simply to aging, or due to aging in conjunction with a degenerative disease. However, it is the stage of the disease that to a greater extent conditions disability and the severity of involvement, and in this case, there was no relationship. There was also no relationship with the first symptom, although a previous study with RVI tasks in PD has shown that if the first symptom was postural instability, the performance of virtual tasks was less well-executed [54]. In our case, and probably due to the short duration of the virtual tasks, this fact had no influence, although a certain relationship was found in the non-faller vs. faller condition, which was normally linked to the progression of the disease. More studies are needed to clarify this topic.

Other studies have proposed multimodal approaches, such as combining voice and image tests to improve the detection of PD patients by means of a smartphone-based application, and by taking into account the impact of some of the symptoms of the disease [59]. In our case, we have tried to analyze the impact of another symptom through the use of a VR tool. The reaction time variables can regularly discriminate falls in PD, suggesting a cut-off point (0.574 s). 

We defined an optimal cut-point value with ROC analysis [60]. The analysis of the ROC curves reported that the variable that best discriminates the risk of suffering a fall in PD patients is that which involves a physical and cognitive component, which in our case was the TUG-Cognitive test score (AUC = 0.85, % sensitivity = 85, % specificity = 90.5) [20]. On the other hand, the proposed IVR task also involved a physical and cognitive component, though its degree of discrimination was lower (AUC = 0.74, % sensitivity = 70, % specificity = 75), maybe because the IVR test incorporates strong multisensory stimulation (visual, auditory and haptic inputs).

### 4.1. Practical Implications

In view of our results, the proposed IVR software could have a dual utility. On the one hand, it could be used to assess response times in functional tasks and, based on this, predict the subject’s risk of falling. On the other, it could be considered a training method to improve these times and thus cognitive aspects and functionality, and also to prevent falls or minimize the injuries that they could cause. Furthermore, our study reinforces the theories that given the bidirectional relationship between gait–cognition and falls, interventions that improve gait may be beneficial for cognition, and vice versa [61,62].

Exercise programs have been shown to be beneficial for improving executive function and increasing gait speed in the elderly [63]. In turn, they have shown how improvements in cognitive aspects such as executive functions are associated with increases in gait speed and muscle strength [64]. PD and Alzheimer’s disease are no exception and, as diseases with a high prevalence in the elderly, the influence of cognitive aspects on the risk of falls has also been extensively studied [65,66,67,68].

### 4.2. Future Research

This study opens the door to future research exploring improvements in balance, function and fall risk in people with PD through reaction time training programs using IVR. As we have discussed, so far these have been carried out with finger tapping tasks, often with desktop computers and a mouse click, involving the movement of a single finger. Our proposal seeks to generate faster but also more functional reactions, since they involve the performance of all of the upper limbs, as well as trunk and head mobility. These functional reactions can be critical in the avoidance of falls and their subsequent injuries. Furthermore, future studies should explore the assessment of whether or not functional RT using IVR is a valid means to predict fall risk and how strongly functional RT is correlated with finger-based RT in PD patients and healthy people.

### 4.3. Limitations

There are some limitations to this study, although the outcomes are promising. The first limitation is that although the sample may be relevant in terms of the number of participants, it may not be wholly representative of the population of patients with Parkinson’s disease and their different profiles (gender, conditions, stage, etc.). A second limitation is that there was only an IVR test, and the conventional computer-based test was not performed to explore the correlation.

## 5. Conclusions

In summary, this study presents a novel test to measure reaction times applied to people with PD in an IVR setting and to train functional reaction times that may contribute to preventing falls. In addition, relationships with functionality, cognitive impairment and fall risk are presented and it is suggested that the reaction times achieved may be predictors of fall risk in PD. In light of this, IVR could be considered a complementary assessment and treatment tool for the health of PD patients.

## Figures and Tables

**Table 1:** Sample selection criteria.

Inclusion Criteria	Exclusion Criteria
Patients diagnosed with PD	An inability to respond to the assessment protocol correctly, as judged by the observing clinician.
Age: 60–80 years	The presence of cardiovascular, pulmonary, or musculoskeletal conditions that would affect the patient’s ability to safely participate in the study, according to the physiotherapist’s judgement.
H&Y: I–III	Severe visual loss, vertigo, epilepsy, psychosis or severe diskinesias that could interfere with the ability to see and perform the IVR test and parkinsonism diagnosis.

H&Y: Hoehn and Yahr scale; PD: Parkinson disease.

**Table 2:** Demographic and clinical characteristics of the sample (fallers and non-fallers).

		All Participants *n* = 26 %/Mean ± SD	Fallers *n* = 10 %/Mean ± SD	Non-Fallers *n* = 16 %/Mean ± SD	Mean Difference (95% CI)	*p*
Age (years)		69.73 ± 6.32	68.40 ± 7.79	70.56 ± 5.33	−3.13–7.45	0.408
Gender (Male)		79.2%	68.8%	70.0%	-	0.946
H&Y Scale		1.54 ± 0.90	1.80 ± 0.79	1.38 ± 0.96	−1.17–0.32	0.252
MMSE Score		26.92 ± 2.64	25.90 ± 2.42	27.56 ± 2.63	−0.46–3.78	0.120
DBS surgical (No)		96.2%	100%	93.8%	-	0.420
First Symptom	Tremor	46.2%	60.0%	37.5%	-	0.728
	Bradykinesia Rigidity	15.4%	10.0%	18.8%	-	
	Postural Instability	11.5%	20.0%	12.5%	-	
	Others	26.9%	10.0%	31.3%	-	

DBS: deep brain stimulation; H&Y: Hoehn and Yahr scale; MMSE: mini mental state exam.

**Table 3:** Sensitivity, specificity, and likelihood ratio (LR) for reaction time and 2 types of timed Up and Go test (TUG).

Variable (Cut-Off Times, s)	AUC (95% CI)	% Sensitivity (95% CI)	% Specificity (95% CI)	Positive LR (95% CI)	Negative LR (95% CI)
Reaction time (0.574)	0.74 (0.54, 0.95)	70 (80, 60)	75 (69, 82)	2.80 (2.4, 3.3)	0.36 (0.2, 0.4)
TUG Test (10.47)	0.80 (0.57, 0.99)	80 (90, 70)	87.5 (81, 94)	6.40 (3.7, 15)	0.15 (0.1, 0.2)
TUG-Cognitive Test (14.23)	0.85 (0.62, 0.99)	85 (90, 80)	90.5 (92, 89)	8.95 (7.27, 11.2)	0.11 (0.1, 0.14)

AUC: area under the curve, CI: confidence interval, TUG-Cognitive: TUG with an added cognitive task, TUG: timed up and go test, s: seconds.

**Table 4:** Sample differences (non-fallers vs. fallers) in reaction times and 2 types of timed Up and Go test (TUG).

	Non-Fallers			Fallers			*p*
	Median	Min	Max	Median	Min	Max	
Reaction time (s)	0.48	0.33	0.846	0.63	0.442	1.13	0.019
TUG test (s)	9.40	6.77	11.06	12.09	7.39	26.00	0.014
TUG-Cognitive test (s)	12.30	8.43	13.86	17.68	14.13	37.17	0.007

TUG: timed up and go test; s: seconds.

**Table 5:** Correlation between reaction time and cognitive, functional status, age and conditions of participants.

		MMSE Score	TUG Test (s)	TUG Cognitive Test (s)	Age (Years)	Non-Fallers (0) vs. Fallers (1)
Reaction time (s)	Spearman’s correlation (rho)	−0.576	0.537	0.454	0.399	0.456
	*p*-value	0.002	0.005	0.020	0.043	0.019

MMSE: mini mental state exam; TUG: timed up and go test.

## Data Availability

The data presented in this study are available upon request from the corresponding author.
